# Zebrafish models of inflammation in hematopoietic development and disease

**DOI:** 10.3389/fcell.2022.955658

**Published:** 2022-07-18

**Authors:** Sarada Ketharnathan, Vinothkumar Rajan, Sergey V. Prykhozhij, Jason N. Berman

**Affiliations:** ^1^ Children’s Hospital of Eastern Ontario Research Institute, Ottawa, ON, Canada; ^2^ Biological Sciences Platform, Sunnybrook Research Institute, Toronto, ON, Canada; ^3^ Departments of Pediatrics and Cellular and Molecular Medicine, University of Ottawa, Ottawa, ON, Canada

**Keywords:** inflammation, hematopoietic stem and progenitor cell (HSPC), zebrafish, interferon, JAK-STAT signaling pathway, bone marrow failure disorders, inflammasome, clonal hematopoiesis

## Abstract

Zebrafish offer an excellent tool for studying the vertebrate hematopoietic system thanks to a highly conserved and rapidly developing hematopoietic program, genetic amenability, optical transparency, and experimental accessibility. Zebrafish studies have contributed to our understanding of hematopoiesis, a complex process regulated by signaling cues, inflammation being crucial among them. Hematopoietic stem cells (HSCs) are multipotent cells producing all the functional blood cells, including immune cells. HSCs respond to inflammation during infection and malignancy by proliferating and producing the blood cells in demand for a specific scenario. We first focus on how inflammation plays a crucial part in steady-state HSC development and describe the critical role of the inflammasome complex in regulating HSC expansion and balanced lineage production. Next, we review zebrafish studies of inflammatory innate immune mechanisms focusing on interferon signaling and the downstream JAK-STAT pathway. We also highlight insights gained from zebrafish models harbouring genetic perturbations in the role of inflammation in hematopoietic disorders such as bone marrow failure, myelodysplastic syndrome, and myeloid leukemia. Indeed, inflammation has been recently identified as a potential driver of clonal hematopoiesis and leukemogenesis, where cells acquire somatic mutations that provide a proliferative advantage in the presence of inflammation. Important insights in this area come from mutant zebrafish studies showing that hematopoietic differentiation can be compromised by epigenetic dysregulation and the aberrant induction of signaling pathways.

## Introduction

Hematopoietic stem cells (HSCs) provide a constant supply of blood cells throughout the lifespan of an organism through their unique properties of self-renewal and multilineage differentiation. Recent advances in single-cell and lineage-reconstruction approaches revealed that HSCs give rise to heterogenous progenitors with different lineage-specific differentiation tendencies called multipotent progenitors (MPPs) (Pietras et al., 2015; Carrelha et al., 2018; Olson et al., 2020). HSCs are typically maintained in a quiescent state within specialized niches in the bone marrow. However, under stress, such as inflammation, they become activated and ramp up myeloid cell production through the increased generation of myeloid-fated MPPs and rewiring of lymphoid-fated MPPs (Pietras et al., 2015; Hérault et al., 2017). Such demand-adapted or emergency hematopoiesis requires robust long-range signaling between pathogen-sensing macrophages and neutrophils in the periphery and bone marrow-resident HSCs to ensure the optimal production of the blood cells necessary to achieve homeostasis. Over the past decade, work from several groups has shown that the same inflammatory signaling pathways that regulate embryonic HSC development also drive emergency hematopoiesis. These include proinflammatory cytokines such as interleukins (ILs), interferons (IFNs), tumor necrosis factor (TNF), and downstream signaling factors such as nuclear factor κB (NFκB) (Hall et al., 2016; Collins et al., 2021; Sugden and North, 2021).

Zebrafish (*Danio rerio*) are increasingly used to model cancer predisposition and blood and immune system disorders, given a highly conserved hematopoietic program detailed elsewhere (Paik and Zon, 2010; Rasighaemi et al., 2015; Kobar et al., 2021). Like in other vertebrates, zebrafish hematopoiesis occurs in two major waves. In the primitive wave, bipotential hemangioblasts give rise to the first hematopoietic (erythrocytes and phagocytes) and endothelial cells. Primitive macrophages and neutrophils specified from the anterior lateral mesoderm acquire phagocytosing capability by 24 hours post-fertilization (hpf). In the definitive HSC-dependent wave, erythroid, myeloid, and B-lymphocytes are produced in the kidney marrow and T-lymphocytes in the thymus. By 4 days post-fertilization (dpf), HSC-derived macrophages colonize definitive tissues. The adaptive immune system starts functioning following the specification of B cells at approximately 21 dpf (Page et al., 2013). Zebrafish neutrophils and macrophages display many morphological and functional similarities with their human counterparts, including the presence of myeloperoxidase-containing granules, production of neutrophil extracellular traps (NETs) in neutrophils (Lieschke et al., 2001; Isles et al., 2021) and the generation of nitric oxide and reactive oxygen species in macrophages (Herbomel et al., 1999; Hermann et al., 2004). Zebrafish offer several advantages compared to other models used to study inflammation, such as mice and human cell lines. External fertilization, transparency of zebrafish embryos, and the availability of transgenic reporter lines that mark various hematopoietic cell types facilitate the study of crucial processes like HSC specification and immune cell migration. Further, the ease of genetic and chemical manipulation has enabled the discovery of intrinsic and extrinsic regulators of hematopoiesis. This review highlights the insights gained from zebrafish models into the role of inflammation in regulating steady-state hematopoiesis and hematopoietic imbalance in hematological disorders.

## Inflammatory signaling in steady-state hematopoiesis

### Sterile inflammation regulates hematopoietic stem cell homeostasis

**FIGURE 1:**
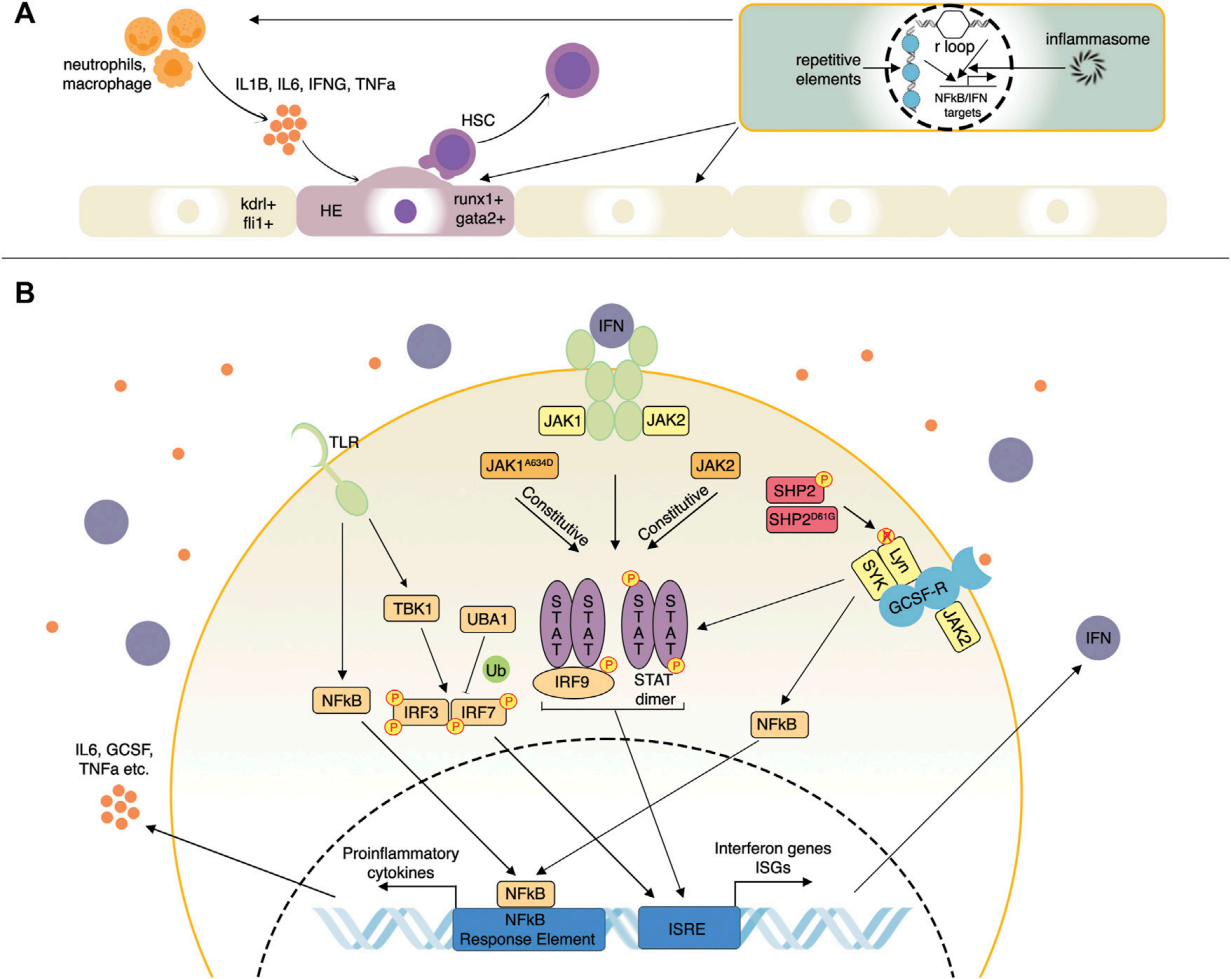
Selected inflammatory pathways affecting hematopoiesis studied in zebrafish. **(A)** Inflammatory influences driving the emergence of hematopoietic stem cells (HSC). Proinflammatory cytokines such as Il1b, Il6, Ifn-γ, and Tnf secreted by neutrophils and macrophages can induce a hemogenic cell fate (runx1+; gata2+) in the vascular endothelium (kdrl+; fli1+). Endogenous innate immune signaling from R-loops, repetitive element transcripts, inflammasomes, and others can promote HSC emergence via NF-kappaB and IFN signaling pathways, as well as several others. **(B)** Proinflammatory cytokine and IFN signaling pathways. Pattern-recognition receptors such as TLR induce complex signaling pathways involving NFkB and TBK1 resulting in IRF3 and IRF7 activation. IRF3/7 are also regulated by ubiquitination factors such as UBA1. IRF3/7 then induce IFN genes and IFN-stimulated genes (ISGs). IFNs then bind their receptors, activating the JAK-STAT pathway that induces additional ISGs more potently. JAK1/2 mutations can result in ligand-independent activation of the JAK-STAT pathway. Mutations of other negative regulatory factors such as SHP2 can stimulate signaling from multiple receptors such as GCSF-R by enhancing the activity of receptor-associated kinases such as Lyn and SYK.

In vertebrate embryos, nascent HSCs emerge from the hemogenic endothelium of the dorsal aorta in the aorta-gonad-mesonephros (AGM) region, where a specialized subset of endothelial cells expressing transcription factors such as *runx1* and *gata2* undergo an endothelial-to-hematopoietic transition (EHT) (Bertrand et al., 2007; Swiers et al., 2013; Kobayashi, 2018) (Figure 1A). Whereas the importance of Tnfα and Notch signaling for embryonic HSC specification has been well-established, the mechanistic basis of this interaction was first illustrated using Tnfα- or Tnfr2-deficient zebrafish (Espín-Palazón et al., 2014). Tnfα produced by primitive neutrophils stimulates NFκB-dependent expression of the Notch ligand, Jag1, in Tnfr2^+^ endothelial cells. Jag1 binds its receptor, Notch1a, on neighboring endothelial cells, where it activates *runx1*, enforcing a hematopoietic cell fate. Other proinflammatory pathways that were identified to positively impact HSC emergence in zebrafish include Tlr4/Myd88/NFκB axis upstream of Notch (He et al., 2015), Ifnγ through the Crfb17 receptor, and Stat3 (Sawamiphak et al., 2014), Il6 through the Il6r receptor (Tie et al., 2019) and Gcsfa/Gcsfb through the Gcsfr receptor (Figure 1B) (Stachura et al., 2013). The primary sources of these signals were primitive macrophages and neutrophils (Espín-Palazón et al., 2014; Tie et al., 2019; Collins et al., 2021) (Figure 1A).

Several recent studies have focused on what induces inflammation during HSC emergence as early embryonic development is considered primarily sterile. Frame et al. (2020) recently demonstrated that metabolic alterations elicit sterile inflammation by activating the NLRP3 inflammasome complex in zebrafish embryos exposed to exogenous glucose. Inflammasomes are multimeric protein complexes of the innate immune system that activate caspases to promote inflammation in response to stimuli like infection or tissue damage. Elevated levels of reactive oxygen species and Hif1α resulting from elevated glucose metabolism induced inflammasome-dependent Il1β production in primitive macrophages, promoting HSC production. In emergency myelopoiesis, Il1β directly regulates the proliferation of myeloid progenitors through the NfκB-Cebpβ signaling network (Wei et al., 2020). However, in a contradictory study, the inflammasome was dispensable for HSC specification but essential for subsequent fate determination and terminal erythroid differentiation. Inflammasome-deficient zebrafish exhibited enhanced erythropoiesis at the expense of myelopoiesis owing to a failure of caspase-dependent inactivation of the erythroid transcription factor, Gata1 (Tyrkalska et al., 2019). Another inducer of HSC production in zebrafish is adenosine. Shear stress generated by blood flow can release ATP, which is quickly converted to adenosine (Yegutkin et al., 2000). Upon binding the A_2b_ adenosine receptor that is enriched in hematopoietic and endothelial cells, adenosine activates the cAMP-protein kinase A pathway, leading to increased Cxcl8 expression. A_2b_ knock-down in zebrafish led to a loss of *cxcl8/cxcr1* signaling, resulting in reduced EHT and diminished HSC output. Certain nucleic acids, such as repetitive element transcripts and R-loops can also stimulate HSC emergence. During EHT, transposable elements expressed in the zebrafish hemogenic endothelium activate retinoic-acid-inducible gene 1-like receptors (RLRs), which are innate immune receptors that trigger NFκB and IFN-mediated inflammatory responses (Jing et al., 2015). Morpholino-induced knock-down of the RLRs Rig-1 or Mda5 in zebrafish impaired HSC production by inhibiting inflammatory signaling (Lefkopoulos et al., 2020). R-loops are RNA:DNA hybrids that form as a natural consequence of transcription when newly transcribed RNA hybridizes to its transiently accessible DNA template. Knock-down of *ddx41*, a DEAD-box helicase involved in clearing R-loops, led to R-loop accumulation and activation of the cyclic GMP-AMP synthase (cGAS)-stimulator of interferon genes (STING) signaling pathway, resulting in enhanced HSC production in zebrafish (Frame and North, 2021; Weinreb et al., 2021). Overall, these studies highlight the importance of inflammation in regulating steady-state hematopoiesis, as well as the power of the zebrafish model in uncovering critical molecular players in HSC development (Figure 1A). These insights will inform the successful production of functional HSCs for treating blood and immune-related disorders.

## Hematopoietic disease models of signaling dysregulation

### Interferon and Janus kinase-signal transducer and activator of transcription signaling axis

Interferons (IFNs) are cytokines produced by multiple immune and non-immune cells upon the detection of microbial or endogenous aberrant RNA and DNA products by pattern recognition receptors (PRR) whose signaling involves IRF3 as a transcriptional activator of IFN genes (Mazewski et al., 2020). IFNs are grouped into three types: type I (IFNα and IFNβ), type II (IFNγ), and type III (IFNλ). All IFNs activate signaling through Janus kinase (JAK)—signal transducer and activator of transcription (STAT) pathway as well as via several other non-canonical pathways (Chen et al., 2017). For example, type I IFNs bind to their receptors to promote activation of JAK1 and TYK2, whose activity results in STAT1 and STAT2 phosphorylation; STAT1/2-IRF9 complex and STAT1 dimers drive the expression of IFN-stimulated genes (ISGs) (Mazewski et al., 2020) (Figure 1B). Type II IFNγ signals by binding to its receptor associated with JAK1 and JAK2, which collectively activate STAT1, leading to its dimerization and transcription factor activity resulting in immediate expression changes and extensive epigenetic reprogramming. IFNγ regulates all branches of the immune system by stimulating lymphocytes, macrophages, as well as other immune cells, and plays a critical proinflammatory role in homeostatic HSC function, as described previously (Ivashkiv, 2018). In addition, IFN signaling molecules are subject to extensive post-translational modifications that control their rapid activation as the first line of protective immunity and subsequent inhibition, thus limiting tissue damage (Chen et al., 2017). This section will highlight the zebrafish studies of IFN/JAK-STAT pathway function and dysregulation in the context of hematopoiesis and blood-related diseases.

Zebrafish are becoming a valuable model for studying the general mechanisms and regulation of IFN signaling. Identifying ISGs is particularly important to expand on the relatively few homologs of known mammalian ISGs. ISGs collectively confer a robust antiviral state in the cells expressing them (Schneider et al., 2014). A study in zebrafish employing IFNφ1 over-expression, chikungunya virus infection, and IFNR (*crbf1*) knock-down identified a group of >400 ISGs (Levraud et al., 2019), proving to be an excellent resource for assessing ISG induction. Multiple innate immune pathways such as cGAS/STING, some TLRs (Toll-like receptors), and RLRs converge on TBK1-mediated activation of IRF3 and IRF7, which are known ISGs that stimulate IFN ligand expression (Figure 1B). IRF3 ubiquitination by different ubiquitin linkages, such as K63 and K48, can promote protein activity and degradation, respectively. IRF3 K63 ubiquitination promotes IRF3 activity, and this modification is opposed by the Otud6b protein, which was first shown in zebrafish (Zhou et al., 2021). Over-expression of Otud6b in zebrafish attenuated induction of viral and chemical IFN signaling, whereas loss of *otud6b* strongly promoted the antiviral response. Moreover, Otud6b physically interacts with Irf3 and Irf7 proteins in zebrafish resulting in suppression of K63 ubiquitination (Zhou et al., 2021). A recent study on genetic screening of patients with systemic inflammation identified a group of males with somatic mutations in the ubiquitin-activating enzyme 1 (UBA1), an X-linked gene encoding an enzyme that catalyzes the first step of ubiquitination (Beck et al., 2020) (Figure 1B). These patients suffered from late-onset severe inflammatory syndromes with diverse clinical features such as chondritis, skin lesions, thromboembolic, and progressive hematologic abnormalities, including macrocytic anemia, thrombocytopenia, and myeloid dyspoiesis. Zebrafish models expressing specific inactivating Uba1 isoforms showed that only loss of *uba1b* that corresponds most closely to the human UBA1 mutations led to a pronounced upregulation of the inflammatory response, including IFN signaling (Beck et al., 2020). Another study of an antiviral response in zebrafish cell lines and embryos demonstrated an upregulation of *uba1* by viruses and poly (I:C) nucleic acids as well as Uba1-mediated suppression of IFN signaling and antiviral responses through IRF3 K48 ubiquitination leading to degradation of IRF3 (Figure 1B) (Chen et al., 2021). In summary, regulatory mechanisms controlling IFN signaling can be targets for pathogenic changes resulting in aberrant signaling activation, and zebrafish mutant disease models can be helpful for fundamental and applied pre-clinical studies.

JAK kinase family consists of JAK1, JAK2, JAK3, and TYK2 kinases, which associate with receptors of at least 30 different cytokines, including IFNs. Such functional versatility can explain their involvement in many inflammatory and neoplastic conditions such as rheumatoid arthritis, ulcerative colitis, Crohn’s disease, hypereosinophilia, myelofibrosis, polycythemia vera, and other myeloproliferative illnesses (Roskoski, 2016). Therefore, extensive efforts are devoted to developing inhibitors targeting several or individual JAK family members. There has been little work on JAK family genes in zebrafish in the context of blood development and inflammation. Studies of *jak2a* in zebrafish employed approaches to study the endogenous gene and over-expression of mutant and fusion versions of the gene. Knock-down of *jak2a* led to a reduction of early hematopoietic progenitors as well as decreased erythroid and myeloid lineages (Ma et al., 2007). By contrast, over-expression of the human JAK2A^V617F^ (Ma et al., 2007) or zebrafish Jak2a^V581F^ (Ma et al., 2009) led to increases in erythroid and myeloid cells, phenocopying polycythemia vera seen in humans, a complex hematological condition with inflammatory features. Ubiquitous overexpression of acute lymphoblastic leukemia (ALL)-derived *tel-jak2a* and chronic myelogenous leukemia (CML)-derived *tel-jak2a* fusions both led to myeloid progenitor expansion, anemia, and thymus expansion. The CML-derived *tel-jak2a* fusion additionally impaired myeloid progenitor differentiation (Onnebo et al., 2012). However, none of the early studies demonstrated if these *jak2a* zebrafish models can be used for understanding the inflammatory aspects of JAK2-mediated diseases, a task likely requiring novel transgenic and gene editing approaches to overcome the limitations of transient reverse genetics. By contrast, *jak1* has been understudied in the hematopoietic context apart from the demonstration that *jak1* mutants fail to develop a thymus (Iwanami et al., 2011). We recently generated transgenic zebrafish lines expressing human JAK1^WT^ and JAK1^A634D^ (Cordeiro-Santanach et al., 2018) to model previously described cases of human hypereosinophilia, where the condition could be improved dramatically by the JAK1/2 inhibitor, ruxolitinib (Del Bel et al., 2017). Whereas JAK1^WT^ expression did not affect zebrafish development, JAK1^A634D^ was toxic, and embryos could only be grown under conditions of ruxolitinib inhibition. Recovered stable JAK1^A634D^ transgenic zebrafish had expanded HSC and myeloid compartments, including allergy-relevant mast cells; Gata1-positive erythroid progenitors were increased, but hemoglobin levels were slightly reduced (Cordeiro-Santanach et al., 2018). RNA sequencing of JAK1^A634D^ human whole blood, induced pluripotent stem cells (iPSC), and transgenic zebrafish revealed a shared core set of dysregulated genes involved in Il4, Il13, and IFN signaling. Indeed, ISGs comprised >20% of all upregulated genes in JAK1^A634D^ zebrafish datasets. Overall, these transgenic zebrafish lines strongly complemented clinical and cell-line data to reveal the role of JAK1^A634D^ and provide the framework for further development of JAK-STAT-related hematopoietic and inflammation models.

## Ribosomopathies, cytopenias, and inflammatory signaling

Ribosomal protein defects (ribosomopathies) frequently result in complex hematological diseases with incompletely understood pathogenic mechanisms. Diamond-Blackfan Anemia (DBA), an inherited bone marrow failure syndrome (BMFS) characterized by multiple hematopoietic and other defects, is caused by mutations in genes encoding structural ribosomal proteins (Rissone and Burgess, 2018). Zebrafish DBA models have been instrumental in understanding the inflammatory mechanisms in this disease. Quantitative PCR analysis of two independent zebrafish DBA models (*rpl11* mutants and *rps19* morphants) showed upregulation of IFN pathway genes, ISGs, *ifi1h*, TLR genes, and proinflammatory cytokine genes (Danilova et al., 2018). Due to the nature of the genetic defects, this proinflammatory innate immune response could indeed be attributed to endogenous factors, such as aberrant RNA components resulting from disrupted ribosome assembly. Whether inflammatory signaling is a side effect or an integral part of the pathogenic mechanism in DBA models has been addressed in a more recent *rpl18* zebrafish mutant study (Chen et al., 2020). Like multiple other ribosomopathy zebrafish models, *rpl18* mutants activate p53 and JAK-STAT pathways. However, suppressing p53 led to only a modest rescue of hematopoiesis, whereas JAK-STAT inhibition was much more effective. By contrast, in other ribosomal protein gene mutants, p53 inhibition was sufficient to restore hematopoiesis (Uechi and Kenmochi, 2019). Thus, the relative importance of anti-inflammatory and anti-p53 treatments will likely remain an active area of study, of which anti-p53 approaches may be less attractive due to possibly elevated cancer risk. The opportunity of exploiting innate immune mechanisms to counteract hematological defects in BMFS was demonstrated in an *rps14* mutant zebrafish model of myelodysplastic syndrome. A TLR7 agonist, imiquimod, was identified through a chemical screen to partially rescue erythropoiesis in *rps14* mutants, morphants, and heterozygous embryos under different conditions (Peña et al., 2021).

Similarly, a zebrafish model of congenital amegakaryocytic thrombocytopenia (CAMT) carrying mutations in the *mpl* gene that encodes the thrombopoietin (Tpo) receptor revealed that the resulting thrombocytopenia is caused by impaired JAK-STAT signaling. Exposure of *mpl*-mutant zebrafish to Il11 bypassed the Tpo-Mpl interaction and activated JAK-STAT to rescue platelet production (Lin et al., 2017). In addition to endogenous inflammatory dysregulation, increased sensitivity to external inflammatory stimuli may accelerate disease in certain BMFS. Zebrafish *rad51* loss-of-function mutants mimic the hypocellular marrow seen in Fanconi anemia, an inherited BMFS characterized by genomic instability and pancytopenia. While *rad51* loss itself did not induce inflammation, it compromised the ability to counteract external inflammatory stress and exacerbated marrow failure in a p53-dependent manner (Botthof et al., 2017). Collectively, these studies demonstrate the importance of inflammation in BMF pathogenesis and the potential for exploiting immunomodulatory therapies to rescue cytopenia and potentially prevent the evolution into clonal hematopoiesis and leukemia.

## Inflammation in clonal hematopoiesis

Clonal hematopoiesis of indeterminate potential (CHIP) is a condition where circulating blood cells have leukemia-associated mutations, albeit without any hematological neoplasm. *DNMT3A*, *TET2*, and *ASXL1* are epigenetic regulators, the most commonly mutated genes in CHIP (Young et al., 2016). Several groups have successfully modeled these mutations in zebrafish, some of which have demonstrated the influence of inflammation on the clonal fitness of mutant cells. We recently published a novel loss-of-function *tet2* zebrafish mutant that exhibits limited hematopoiesis, reduced activation of inflammatory pathways, and elevated p53 protein levels at a steady state. We hypothesized that the reduced inflammation is a protective mechanism and that *tet2*-mutant cells would exhibit a proliferative advantage upon proinflammatory signaling activation. Indeed, activation of proinflammatory pathways provided *tet2*- mutant blood cells with a proliferative advantage and an increase in differentiation block (Rajan et al., 2021). Similar results were observed in *Dnmt3a* models with chronic exposure to IFN gamma (Hormaechea-Agulla et al., 2021).

In an elegant study recently published by the Zon lab, lineage-tracing coupled with mosaic mutagenesis in zebrafish was used to study the clonal evolution of common CHIP mutations. Longitudinal cell tracking by retro-orbital bleeding of the fish allowed them to look at clonal competition and fitness conferred by a particular mutation (Avagyan et al., 2021). Unlike *tet2* and *dnmt3a* mutations, *asxl1* mutations regulated inflammatory signals in a cell-autonomous manner. Mutant neutrophils and macrophages secreted inflammatory cytokines, whereas HSCs and progenitor cells expressed anti-inflammatory genes. Ablation of *nr4a1*, an immunomodulator, rescued this effect by suppressing the clonal dominance of *asxl1* cells. An elevated inflammatory cytokine signature has also been observed in germline *asxl1* mutant zebrafish (Fang et al., 2021). These mutants displayed neutrophil deficiency and leukemic progression, with a small percentage of fish developing a CMML-like or AML-like phenotype around 1 year of age.

## Inflammation and leukemia

We and others have created zebrafish leukemia models to help understand disease mechanisms and uncover potential therapeutic targets (Langenau et al., 2003; Chen et al., 2007; Forrester et al., 2011; Deveau et al., 2015). Since the association between inflammation and leukemia was only recently identified and due to the absence of an aged leukemic zebrafish model, studies are limited. A gain-of-function mutation of *SHP2* is frequently observed in sporadic juvenile myelomonocytic leukemia (JMML) and JMML-like-MPNs (Solman et al., 2022). SHP2, when phosphorylated, activates receptor-associated kinases like Lyn kinase; by dephosphorylating them, a gain-of-function mutation of SHP2 makes the dephosphorylation and activation constitutive (Figure 1B). Zebrafish carrying a point mutation in Shp2^*D61G*^ mimicked the myeloproliferative phenotype seen in humans. Further, when blood cells from larvae were subjected to single-cell sequencing, it was found that the mutant monocyte population expressed high levels of inflammatory genes. Particularly *gcsfa, gcsfb, il1b, irg1,* and *nfkbiaa* were highly expressed in Shp2^*D61G*^ monocytes compared to normal cells. Treatment with anti-inflammatory drugs such as dexamethasone reversed the aberrant proliferation (Solman et al., 2022).

The zebrafish disease modeling field has been very dependent on transgenic technologies for gain-of-function and on morpholinos for loss-of-function disease model generation. However, morpholinos are short-lived and limit our ability to study the effects of genetic loss of function in an aging-related inflammatory milieu. Given the renaissance of gene editing technologies, there are now ample options to develop more precise leukemia models that are based upon stable loss-of-function and point mutations. An alternative option is to mimic the inflammatory environment by artificially providing inflammatory cytokines like we did using our *tet2* loss-of-function model to study the effect of this mutation during emergency hematopoiesis (Rajan et al., 2021). Overall, we find ourselves in a time where tools are becoming more widely available to interrogate the role of inflammation in the context of preleukemia to leukemia evolution, leukemia exacerbation and identify therapeutic anti-inflammatory molecules.

## Conclusion

Inflammatory signaling lies at the core of many developmental, physiological, and pathological processes of the hematopoietic system. Animal model studies of this system are critical to understanding the dynamics of these processes and the underlying *in vivo* mechanisms. The zebrafish is an effective model system to facilitate our understanding of how inflammation impacts hematopoiesis and can serve as an *in vivo* platform for pre-clinical studies. This article reviewed zebrafish studies examining how inflammation regulates the homeostasis of HSCs, the function and regulation of the IFN-JAK-STAT signaling axis, and the roles of inflammatory signaling in ribosomopathies. We also touched on zebrafish models of epigenetic and signaling abnormalities implicated in clonal hematopoiesis and leukemia. Collectively, these studies support the translational relevance of *in vivo* zebrafish models and highlight how both disease pathogenesis and potential treatment opportunities in several hematopoietic disorders can be best understood by focusing on inflammatory mechanisms.
